# Kinetics and inhibition studies of the L205R mutant of cAMP‐dependent protein kinase involved in Cushing's syndrome

**DOI:** 10.1002/2211-5463.12396

**Published:** 2018-03-11

**Authors:** Nicole M. Luzi, Charles E. Lyons, Darrell L. Peterson, Keith C. Ellis

**Affiliations:** ^1^ Department of Medicinal Chemistry School of Pharmacy Virginia Commonwealth University Richmond VA USA; ^2^ Massey Cancer Center Virginia Commonwealth University Richmond VA USA; ^3^ Department of Biochemistry and Molecular Biology School of Medicine Virginia Commonwealth University Richmond VA USA; ^4^ Institute for Structural Biology, Drug Discovery, and Development Virginia Commonwealth University Richmond VA USA

**Keywords:** ACTH‐independent Cushing's syndrome, cAMP‐dependent protein kinase, enzyme inhibition, kinetics, L205R‐PKA

## Abstract

Overproduction of cortisol by the hypothalamus–pituitary–adrenal hormone system results in the clinical disorder known as Cushing's syndrome. Genomics studies have identified a key mutation (L205R) in the α‐isoform of the catalytic subunit of cAMP‐dependent protein kinase (PKACα) in adrenal adenomas of patients with adrenocorticotropic hormone‐independent Cushing's syndrome. Here, we conducted kinetics and inhibition studies on the L205R‐PKACα mutant. We have found that the L205R mutation affects the kinetics of both Kemptide and ATP as substrates, decreasing the catalytic efficiency (*k*_cat_/*K*_M_) for each substrate by 12‐fold and 4.5‐fold, respectively. We have also determined the IC_50_ and *K*_i_ for the peptide substrate‐competitive inhibitor PKI(5–24) and the ATP‐competitive inhibitor H89. The L205R mutation had no effect on the potency of H89, but causes a > 250‐fold loss in potency for PKI(5–24). Collectively, these data provide insights for the development of L205R‐PKACα inhibitors as potential therapeutics.

**Abbreviations**
ACTH: adrenocorticotropic hormone
H89: *N*‐[2‐(*p*‐bromocinnamylamino)ethyl]‐5‐isoquinolinesulfonamide
Kemptide: the PKACα peptide substrate with the sequence H_2_N‐Leu‐Arg‐Arg‐Ala‐Ser‐Leu‐Gly‐OH
MAB: methyl aminobutyric acid
MC_2_R: melanocortin 2 G‐protein‐coupled receptor
PKACα: α‐isoform of the catalytic subunit of cAMP‐dependent protein kinase
PKI(5–24): cAMP‐dependent protein kinase inhibitor, residues 5–24
*PRKACA*: gene encoding cAMP‐dependent protein kinase catalytic subunit alpha
Rh: rhodamine B

Cushing's syndrome is a clinical disorder caused by the overproduction of cortisol by the hypothalamus–pituitary–adrenal hormone system 1. Symptoms of Cushing's syndrome include central obesity, buffalo hump, moon face, and striae, which together form a metabolic syndrome that can cause or worsen the effects of hypertension, heart disease, and diabetes, and lead to morbidity and mortality 1. Pathophysiologically, Cushing's syndrome can be caused by pituitary adenomas that release unregulated amounts of ACTH hormone (ACTH‐dependent Cushing's syndrome), adrenal adenomas that release unregulated amounts of cortisol directly (ACTH‐independent Cushing's syndrome), and ectopic tumors outside the hypothalamus–pituitary–adrenal axis that produce ACTH 1.

Recent genomics studies of patients with ACTH‐independent Cushing's syndrome revealed that ~ 40% of the adrenal adenomas from these patients carried a T617G mutation in the *PRKACA* gene, which encodes for the α‐isoform of the catalytic subunit of cAMP‐dependent protein kinase (PKACα) 2, 3, 4, 5. Under normal physiological conditions, the ACTH peptide hormone binds to and activates the melanocortin 2 receptor (MC_2_R) in the adrenal cortex; MC_2_R is a G_s_‐coupled receptor that, when activated, elevates cAMP levels and activates PKACα 6. The somatic mutation in the *PRKACA* gene results in the expression of a mutant form of PKACα, where Leu205 has been mutated to Arg. The L205R‐PKACα mutation leads to loss of binding of the PKA regulatory subunits (PKARIα/β and PKARIIα/β) 2, 3, 5, 7, 8, loss of sensitivity to cAMP signaling 2, 5, 7, constitutive activation of the mutant PKACα protein 2, 4, 5, and unregulated phosphorylation of PKACα substrates 3, 4, 5, presumably including those involved in downstream cortisol biosynthesis.

Structural biology has given some insights as to how the single‐point mutation causes the disruption in PKACα regulation. In the wt‐PKACα cocrystal structure with the PKI(5–24) inhibitor peptide and ATP 9, 10, Leu205 is part of a group of hydrophobic residues (Leu198, Gly200, Pro202, and Leu205) that form the P+1‐binding pocket (Fig. 1A), an important site for substrate, regulatory subunit, and inhibitor recognition. The hydrophobic side chain of Ile22 of PKI(5–24) binds in the P+1‐binding site, creating a positive binding interaction and contributing to overall inhibitor binding. In the cocrystal structure of the L205R‐PKACα mutant with PKI(5–24) and ATP (Fig. 1B) 10, the mutation of Leu205 to Arg results in the larger Arg205 residue occupying the P + 1 pocket (now defined as Leu198, Gly200, Pro202, and Arg205), the binding pocket normally occupied by Ile22 of PKI. The L205R mutation prevents Ile22 of PKI from binding in the P + 1 pocket and causes a change in binding conformation of the C‐terminal end of PKI(5–24). The mutant structure offers an explanation for the lack of binding by the regulatory subunits and predicts a lack of inhibition of L205R‐PKACα by PKI, as both of these require a hydrophobic binding interaction in the P + 1 pocket of wt‐PKACα for regulation or inhibition.

**Figure 1:**
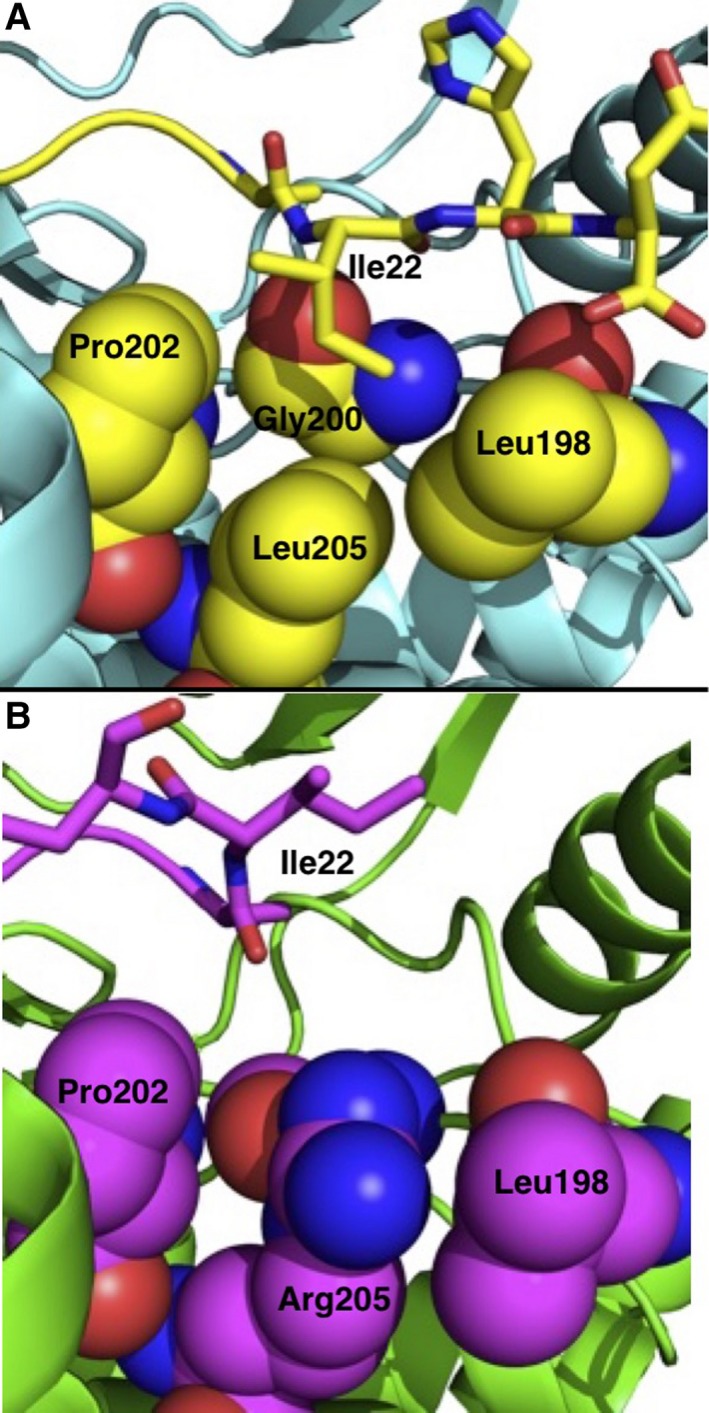
Crystal structures of wt‐PKACα (A) and L205R‐PKACα (B). (A) Crystal structure of wt‐PKACα (Cyan) and PKI (Yellow) with P + 1 pocket residues shown as space‐filling models (PDBID: http://www.rcsb.org/pdb/search/structidSearch.do?structureId=4WB5). (B) Crystal structure of L205R‐PKACα (Green) and PKI (Magenta) with P + 1 pocket residues shown as space‐filling models (PDBID: http://www.rcsb.org/pdb/search/structidSearch.do?structureId=4WB6). For each structure, P + 1 pocket residues are colored the same as PKI.

While the structure of the L205R‐PKACα mutant 10 and the overall effects of the mutation in a cellular environment 2, 3, 4, 5, 7, 8 have been reported, detailed studies of the enzymology of the mutant have not. Measurement of the specific activity of the L205R‐PKACα mutant from cell lysates 2, 4, 5, 7 and purified recombinant protein production 10 have been reported, but a full analysis of the kinetics parameters has not been performed. In addition, the sensitivity of the L205R‐PKACα mutant to known wt‐PKACα inhibitors has been measured in cell lysates at fixed inhibitor concentrations 4, 5, but a study to determine how the L205R mutation affects the IC_50_ and *K*_i_ of these inhibitors has not been carried out. Here, we report a kinetics and inhibition study to fully characterize the enzymology of the L205R‐PKACα mutant and determine the effect of the mutation on enzyme function. These studies are an important first step toward investigating L205R‐PKACα as a therapeutic molecular target for the treatment of ACTH‐independent Cushing's syndrome.

## Materials and methods

### Reagents

All assay reagents, including buffer salts, ATP, and MgCl_2,_ were of molecular biology grade and purchased from Sigma‐Aldrich (St. Louis, MO, USA). H89 was purchased from Selleck Chem (Houston, TX, USA). PKI(5–24) was purchased from Alfa Aesar (Tewksbury, MA, USA). HPLC‐grade solvents were purchased from VWR (Radnor, PA, USA).

### Production of recombinant PKACα kinases

The plasmid for the expression of recombinant human L205R‐PKACα was prepared by GenScript (Piscataway, NJ, USA) using a combination of gene synthesis and molecular biology (see Fig. S1 for gene insert sequence, full plasmid sequence, and resulting protein sequence). First, a protein sequence for full‐length recombinant L205R‐PKACα (Gly1 to Phe350) was designed to include an N‐terminal (His)_6_ tag followed immediately by a TEV cleavage site (ENLYFQ/G), where the remaining glycine residue left after TEV cleavage became the first glycine in the processed protein sequence. To the codons for this sequence (based on human *PRKACA* gene with T617G mutation) were added a 5′‐NdeI restriction site (which includes a start codon), two stop codons, and a 3′‐HindIII restriction site. This entire gene insert was synthesized, cloned into the pET‐17b vector, and the vector sequenced to confirm the correct incorporation of the insert. To express L205R‐PKACα, BL21(DE3)‐competent *Escherichia coli* cells were transformed with the plasmid described above, selecting with ampicillin (50 mg·L^−1^). Bacterial cells were grown in autoinduction media at 37 °C for 16 h. Following growth, cells were spun down to a pellet at 9000 ***g*** for 10 min, lysed by a single pass through an Avestin Emulsiflex (Avestin, Ottawa, ON, Canada) homogenizer operating at 25 000 psi into buffer (25 mm Tris/HCl (pH 8), 300 mm NaCl, and 10 mm imidazole), allowed to equilibrate for 30 min at ambient temperature, and debris removed by centrifugation at 48 000 ***g*** for 30 min. The soluble fraction was then loaded onto a Ni‐NTA column and washed with 5 column volumes of the loading buffer, and pure (His)_6_‐L205R‐PKACα was eluted with 100 mm imidazole. The purified protein was then treated with TEV protease (1 wt%; 1 mg TEV protease per 100 mg L205R‐PKACα) overnight at room temperature to remove the (His)_6_ tag. Following TEV cleavage, the kinase was dialyzed into storage buffer (25 mm sodium phosphate buffer with 10% glycerol and 1 mm DTT), aliquoted, and stored at −80 °C until use. To verify that the recombinant kinase protein contains the L205R point mutation, a trypsin digest was performed and the fragments analyzed LC/MS/MS experiment using direct injection on a LCQ DecaXP Max mass spectrometer (ThermoScientific, Waltham, MA, USA). The fragment containing R205 was identified (^195^TWTLCGTPEY**R** ^205^, calculated: 1327.49 Da; found: 1327.16 Da), confirming installation of the mutation. wt‐PKACα was produced and purified as previously reported 11 in parallel with the L205R‐PKACα mutant.

### Kinetics assay

Kinetics assays were performed using an HPLC‐Vis assay with a rhodamine‐labeled Kemptide analogue (Rh‐MAB‐Kemptide), which we have reported elsewhere 11. Assays were performed in 96‐well plates at 25 °C with a total reaction volume of 200 μL. For determination of kinetics parameters for Kemptide peptide substrate, kinase reactions contained L205R‐PKACα (2 nm), ATP (1 mm), MgCl_2_ (10 mm), and Rh‐MAB‐Kemptide (200–12.5 μm, twofold dilutions) in 50 mm MOPS (pH 7.4, adjusted with NaOH). Reactions were initiated by the addition of the Rh‐MAB‐Kemptide analogue, run for 15 min, and then quenched with 5% phosphoric acid (100 μL). For determination of kinetics parameters for ATP, kinase reactions contained L205R‐PKACα (4 nm), Rh‐MAB‐Kemptide (25 μm), MgCl_2_ (10 mm), and ATP (400–6.25 μm, twofold dilutions) in 50 mm MOPS (pH 7.4, adjusted with NaOH). Reactions were initiated by the addition of ATP, run for 15 min, and then quenched with 5% phosphoric acid (100 μL). Negative controls that consisted of kinase reactions where buffer was added in place of active kinase enzyme were included in all assay runs. All kinase reactions were carried out in triplicate for each concentration of Rh‐MAB‐Kemptide or ATP. The 96‐well plate was then loaded into the HPLC autosampler (Agilent, Santa Clara, CA, USA), and each kinase reaction analyzed by the HPLC method described below. The volume of the injection was varied from 5 to 100 μL such that 0.5 nmoles of chromophore‐labeled peptide was loaded and analyzed for each kinase reaction. Following HPLC analysis, the % phosphorylation was determined for each Rh‐MAB‐Kemptide or ATP concentration by integrating the area of the peaks for the substrate and product at 560 nm. % Phosphorylation data were converted to velocities and plotted against the concentrations of the substrate. Under the kinase reaction conditions above, product conversions were ≤11% at substrate concentrations equal to *K*_M_, ensuring that the experiments were run under initial velocity conditions. Curve fitting and the determination of kinetics parameters were performed in prism 7 (GraphPad, La Jolla, CA, USA) using nonlinear regression to the Michaelis–Menten equation. Three experimental replicates were carried out for each curve, and the mean and SEM for each kinetic parameter were determined. For comparison, wt‐PKACα replicates were performed in parallel on the same 96‐well plates with technical and experimental replicates for the L205R mutant as reported elsewhere 11.

### Inhibition assay

Inhibition assays were performed in 96‐well plates at 25 °C with a total reaction volume of 200 μL. Kinase reactions contained L205R‐PKACα (4 nm), ATP (25 μm), MgCl_2_ (10 mm), Rh‐MAB‐Kemptide (30 μm), inhibitor (H89, 16 μm–0.015 nm or PKI(5–24), 62.5 μm–0.24 nm; fourfold dilutions) in 50 mm MOPS (pH 7.4, adjusted with NaOH). For H89, reactions were initiated by the addition of ATP after a 10‐min preincubation of inhibitor with L205R‐PKACα and the other reaction components. For PKI(5–24), reactions were initiated by the addition of Rh‐MAB‐Kemptide after a 10‐min preincubation of inhibitor with L205R‐PKACα and the other reaction components. Reactions were run for 40 min and then quenched with 5% phosphoric acid (100 μL). Kinase reactions were carried out in duplicate for each concentration of each inhibitor. Negative controls that consisted of kinase reactions where buffer was added in place of inhibitor were included in all assay runs. The 96‐well plate was then loaded into the HPLC autosampler and each kinase reaction analyzed by the HPLC method described below (80 μL injection volume, 0.5 nmoles of chromophore‐labeled peptide). Following HPLC analysis, the % phosphorylation was determined for each inhibitor concentration by integrating the area of the peaks for the substrate and product at 560 nm. % Phosphorylation data were normalized to the control well without inhibitor, converted to % inhibition, and plotted against the log of the concentrations of the inhibitor. Curve fitting and the determination of the IC_50_ were performed in prism 7 (GraphPad) using nonlinear regression to the four‐parameter log(inhibitor) versus response equation. *K*_i_'s were calculated using the Cheng–Prusoff equation and the *K*_M_ for Rh‐MAB‐Kemptide or ATP with L205R‐PKACα (Table 1). Three experimental replicates were carried out for each curve, and the mean and SEM for the IC_50_ and *K*_i_ for each inhibitor were determined. For comparison, wt‐PKACα replicates were performed in parallel on the same 96‐well plates with technical and experimental replicates for the L205R mutant as reported elsewhere 11.

**Table 1:** Kinetics parameters for the L205R‐PKACα mutant

	L205R‐PKACα	wt‐PKACα^a^	Fold change^b^
Rh‐MAB‐Kemptide			
*K*_M_ (μm)	60.9 ± 8.4	9.74 ± 0.88	+6.3
*V*_max_ (μmol·min^−1^·mg^−1^)	11.2 ± 1.7	21.7 ± 3.4	−1.9
*k*_cat_ (min^−1^)	450 ± 70	870 ± 138	−1.9
*k*_cat_/*K*_M_ (μm ^−1^·min^−1^)	7.38 ± 0.52	90.7 ± 22.7	−12.3
ATP			
*K*_M_ (μm)	27.6 ± 4.5	16.9 ± 1.3	+1.6
*V*_max_ (μmol·min^−1^·mg^−1^)	3.23 ± 0.52	9.04 ± 0.65	−2.8
*k*_cat_ (min^−1^)	129 ± 21	362 ± 26	−2.8
*k*_cat_/*K*_M_ (μm ^−1^·min^−1^)	4.76 ± 1.09	21.4 ± 1.2	−4.5

^a^ Kinetics data from Reference 11. ^b^ Positive (+) fold changes represent an increase in the value of the kinetics parameter for the L205R‐PKACα mutant relative to wt‐PKACα; negative (−) fold changes represent a decrease in the value of the kinetics parameter for the L205R‐PKACα mutant relative to wt‐PKACα.

### HPLC assay method

All HPLC experiments were carried out on an Agilent LC 1200 system with a degasser, quaternary pump, vial and well plate autosampler, column heater, and diode array detector. All data were collected and processed with Agilent chemstation software. In all HPLC runs, absorbance was monitored at 560, 550, 280, and 210 nm. For kinetics and inhibition assays, the system was operated in analytical mode using a Restek Ultra C18 3 μm, 2.1 × 100 mm column and a flow rate of 0.3 mL·min^−1^. Assays were analyzed using a gradient of water with 0.1% formic acid (Solvent A) and acetonitrile with 0.1% formic acid (Solvent B); gradient: 0–0.5 min, 5% B; 0.5–9.0 min, 5–77% B; 9.0–9.5 min, 77% B; 9.5–10 min, 77–5% B; 10–13 min, 5% B. % Phosphorylation was determined by integrating both the substrate and product peaks measured at 560 nm.

## Results

### Expression and purification of the L205R‐PKACα mutant

We began our work by designing and synthesizing plasmids for the expression of L205R‐PKACα using a combination of gene synthesis and molecular biology. Our expression plasmids each included an N‐terminal (His)_6_ tag for purification followed by a TEV cleavage site blunt against the first residue of the kinase. In our initial plasmid, we optimized the codons for the *PRKACA* gene, including the T617G mutation, for expression in *E. coli* (data not shown). However, upon growing cultures for protein production with this codon‐optimized plasmid, we observed that bacterial growth was significantly impaired compared to bacterial growth in previous protein production of wt‐PKACα using a non‐codon‐optimized plasmid 12. We hypothesized that expression of large amounts of L205R‐PKACα from use of the codon‐optimized plasmid might be resulting in toxicity to the bacteria. To address this issue, we prepared a second plasmid of the same design, but with endogenous human codons for *PRKACA* and the observed T617G mutation (see Materials and methods and Fig. S1). With this plasmid, bacterial growth in protein production was restored to expected levels. Following lysis, purification, and cleavage with TEV protease, we were able to produce the L205R‐PKACα mutant in quantities of ~ 5 mg·mL^−1^ of culture.

### Kinetics studies

With L205R‐PKACα protein in hand, we next turned to studying the kinetics of the mutant kinase with Kemptide peptide substrate and ATP. For the kinetics work, we utilized an HPLC‐Vis endpoint assay with a rhodamine B‐labeled Kemptide analogue (Rh‐MAB‐Kemptide), which we have reported elsewhere 11. This assay separates and directly quantifies both substrate and phosphorylated product.

**Figure 2:**
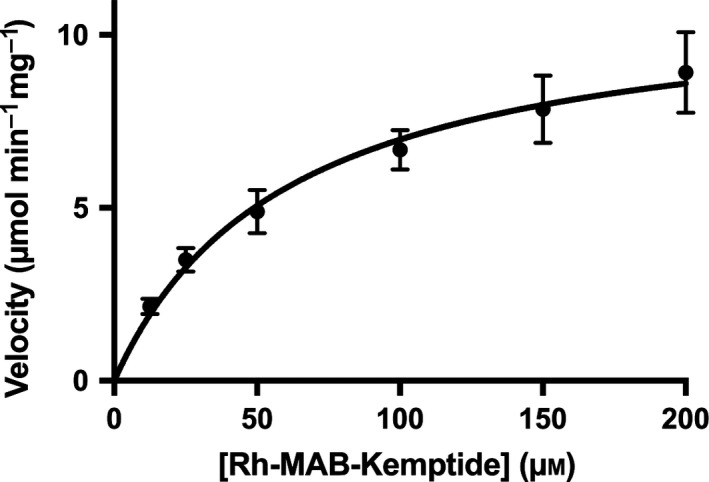
Kinetic analysis of the phosphorylation of Rh‐MAB‐Kemptide by the L205R‐PKACα mutant at a fixed concentration of ATP for determination of *K*_M,Kemptide_ and other kinetic parameters. Similar data for wt‐PKACα can be found in reference 11. The range of values on the graph axes for the kinetics analysis of Rh‐MAB‐Kemptide (this figure) and ATP (Fig. 3) are different.

To determine the effect of the mutation on kinetics with Kemptide, we first ran a series of kinase reactions with varying concentrations of Rh‐MAB‐Kemptide at a fixed concentration of ATP, in order to determine the kinetic parameters for Kemptide (see Materials and methods). Following kinase reactions, percent phosphorylation data were transformed to velocities, and the kinetics parameters were determined by fitting the data to the Michaelis–Menten equation (Fig. 2, Table 1). The *K*_M,Kemptide_ for the L205R‐PKACα mutant (60.9 ± 8.4 μm) was found to be ~ 6‐fold higher than the *K*_M,Kemptide_ for wt‐PKACα (9.74 ± 0.88 μm), likely due to impaired binding of the hydrophobic Leu6 residue in Kemptide to the mutated P + 1 biding site in L205R‐PKACα, resulting in a lower binding affinity of Kemptide for the mutant versus wt‐PKACα. Both the *V*_max,Kemptide_ (11.2 ± 1.7 μmol·min^−1^·mg^−1^) and the *k*_cat,Kemptide_ (450 ± 70 min^−1^) for the mutant were found to be ~ 2‐fold lower than *V*_max,Kemptide_ (21.7 ± 3.4 μmol·min^−1^·mg^−1^) and the *k*_cat,Kemptide_ (870 ± 138 min^−1^) for wt‐PKACα, demonstrating that the mutation slows the overall phosphotransfer chemical reaction. Determination of *k*_cat_/*K*_M_ for Kemptide with the L205R‐mutant (7.38 ± 0.52 μm ^−1^·min^−1^) shows that the catalytic efficiency of the mutant is ~ 12‐fold lower than the *k*_cat_/*K*_M_ for Kemptide with wt‐PKACα (90.7 ± 22.7 μm ^−1^·min^−1^), demonstrating that the L205R mutant has a significantly lower specificity for Kemptide than the wild‐type kinase.

**Figure 3:**
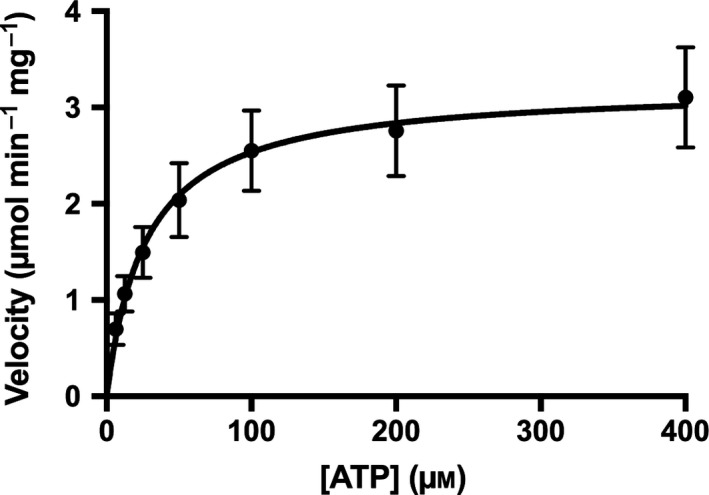
Kinetic analysis of the phosphorylation of Rh‐MAB‐Kemptide by the L205R‐PKACα mutant at varying ATP concentrations for determination of *K*_M,ATP_ and other kinetic parameters. Similar data for wt‐PKACα can be found in reference 11. The range of values on the graph axes for the kinetics analysis of Rh‐MAB‐Kemptide (Fig. 2) and ATP (this figure) are different.

We next turned to determining whether the L205R mutation, which occurs in the peptide substrate‐binding site, has any effect on ATP kinetics (Fig. 3, Table 1). With ATP as a substrate, we observed the same trends that are seen in the Kemptide kinetic parameters, but with a lower magnitude of the effect. The *K*_M,ATP_ for the L205R‐PKACα mutant (27.6 ± 4.5 μm) was found to be higher than the *K*_M,ATP_ for wt‐PKACα (16.9 ± 1.3 μm), but only by a factor of ~ 1.6‐fold. Both the *V*_max,ATP_ (3.23 ± 0.52 μmol·min^−1^·mg^−1^) and the *k*_cat,ATP_ (129 ± 21 min^−1^) for the mutant were found to be lower than *V*_max,ATP_ (9.04 ± 0.33 μmol·min^−1^·mg^−1^) and the *k*_cat,ATP_ (362 ± 26 min^−1^) for wt‐PKACα by 2.8‐fold. Finally, the *k*_cat_/*K*_M_ for ATP with the L205R‐mutant (4.76 ± 1.09 μm ^−1^·min^−1^) was found to be lower than the *k*_cat_/*K*_M_ for ATP with wt‐PKACα (21.4 ± 1.2 μm ^−1^·min^−1^) by a factor of 4.5‐fold.

Collectively, these data demonstrate that the L205R mutation has effects on all of the kinetics parameters, especially the catalytic efficiency (*k*_cat_/*K*_M_), for both Kemptide and ATP despite the fact that the point mutation only occurs in the peptide substrate‐binding site.

### Inhibition studies

**Figure 4:**
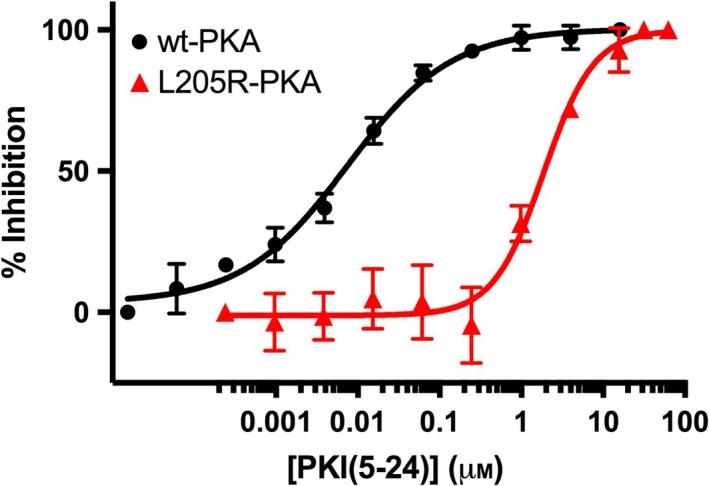
Inhibition of wt‐PKACα (black circles) and the L205R‐PKACα mutant (red triangles) by PKI(5–24). The inhibitor concentration ranges for PKI(5–24) (this figure) and H89 (Fig. 5) are different.

**Figure 5:**
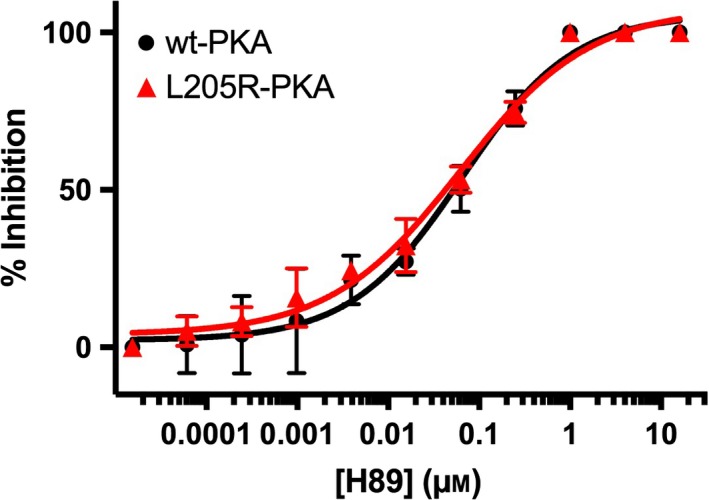
Inhibition of wt‐PKACα (black circles) and the L205R‐PKACα mutant (red triangles) by H89. The inhibitor concentration ranges for PKI(5–24) (Fig. 4) and H89 (this figure) are different.

Having characterized the kinetics of the L205R mutant with both peptide substrate and ATP, we turned to determining the sensitivity of the L205R mutant to known wt‐PKACα inhibitors. We first determined the ability of the peptide substrate‐competitive inhibitor PKI(5–24) to inhibit the phosphotransferase function of the L205R mutant (Fig. 4) in our HPLC‐Vis inhibition assay. As seen in Table 2, the single L205R point mutation has a significant effect on inhibition by PKI(5–24). The IC_50_ (1870 ± 206 nm) and *K*_i_ (1250 ± 138 nm) of PKI(5–24) with the L205R mutant were both found to be > 250‐fold higher than the IC_50_ (7.4 ± 1.2 nm) and *K*_i_ (3.7 ± 0.6 nm) for wt‐PKACα. This is a remarkable decrease in inhibition of the mutant by the simple disruption of binding of one of the twenty amino acids of the inhibitor and underscores the importance of binding to the P+1‐binding pocket for potent inhibition. We then determined the ability of the ATP‐competitive inhibitor H89 to inhibit the mutant (Fig. 5). Unlike PKI(5–24), the L205R mutation seems to have no effect on inhibition of phosphotransferase function by H89. The IC_50_ (60.6 ± 8.4 nm) and *K*_i_ (27.8 ± 5.7 nm) for H89 with the L205R mutant were effectively identical to the IC_50_ (63.3 ± 4.5 nm) and *K*_i_ (25.5 ± 1.8 nm) with wt‐PKACα (Table 2).

**Table 2:** Data for inhibition of the L205R‐PKACα mutant by wt‐PKACα inhibitors

	L205R‐PKACα	wt‐PKACα^a^	Fold change^b^
PKI(5–24)^c^			
IC_50_ (nm)	1870 ± 206	7.4 ± 1.2	253
*K*_i_ (nm)^d^	1250 ± 138	3.7 ± 0.6	338
H89			
IC_50_ (nm)	60.6 ± 8.4	63.3 ± 4.5	0.96
*K*_i_ (nm)^d^	27.8 ± 5.7	25.5 ± 1.8	1.1

^a^Inhibition data from Reference 11. ^b^Fold changes represent the change in the value of the inhibition parameter for the L205R‐PKACα mutant relative to wt‐PKACα. ^c^Sequence for PKI(5–24): H_2_N‐TTYADFIASGRTGRRNAIHD‐COOH. ^d^ *K*_i_'s were calculated using Cheng–Prusoff equation and the *K*_M_'s from Table 1.

## Discussion

This work reports the first study that determines the fundamental kinetics parameters for the L205R‐PKACα mutant kinase with both Kemptide and ATP substrates. We have found that the *K*_M,Kemptide_ for the L205R‐PKACα mutant is ~ 6‐fold higher than for wt‐PKACα. *K*_M_ is generally regarded as a measure of the affinity (*K*_d_) of a substrate for the enzyme; this is an assumption, however, as the expression for *K*_M_ [(*k*_−1_ + *k*_2_)/*k*_1_] contains both the *K*_d_ and the first‐order rate constant for conversion of the [E]·[S] complex to product. In this case, the increase in *K*_M_ indicates that the L205R mutation in the P+1‐binding pocket of the mutant leads to an ~ 6‐fold loss in affinity, due to the loss of the productive binding interaction of residue Leu6 of Kemptide with the P + 1 pocket. The turnover number for the Kemptide substrate (*k*_cat,Kemptide_) was found to be lower for the L205R mutant than for wt‐PKACα by ~ 2‐fold, showing that the rate of catalysis for the mutant is impaired. Previous work with the L205R‐PKACα mutant by Lee and coworkers using live‐cell FRET also showed a reduced catalytic activity with an AKAR4 reporter protein 8, presumably due to loss of the key binding interaction between Leu476 of AKAR4 with the P + 1 pocket of the L205R mutant. Finally, an examination of *k*_cat_/*K*_M_ for Kemptide with the mutant and wt‐PKACα shows a > 12‐fold decrease in the catalytic efficiency. In this case, the decrease in the affinity (*K*_M,Kemptide_) and the turnover number (*k*_cat,Kemptide_) appear to reinforce each other, leading to the large decrease in *k*_cat_/*K*_M_ for Kemptide. The catalytic efficiency reflects the specificity of each enzyme for Kemptide and shows that the L205R mutation leads to the > 12‐fold decrease in specificity versus wt‐PKACα. This is in agreement with recent work by Schwartz and coworkers, demonstrating that the L205R mutation changes the substrate specificity of the kinase and that the L205R mutant more readily phosphorylates substrates with acidic residues in the P + 1 position 13. The catalytic efficiency can also be viewed as the second‐order rate constant and the probability that the [E]·[S] complex will be converted to product. These data support a model for the L205R‐PKACA mutant where the [E]·[S] complex is either dissociating faster than it is being converted to product or that if it is converted to product, catalysis is slower. Our work, taken together with that of Lee 8 and Schwartz 13, also suggests that the L205R‐PKACα mutation causes ACTH‐independent Cushing's syndrome through a change of substrate specificity and not simply constitutive activation of the L205R gain‐of‐function mutant kinase. This change in substrate specificity for the L205R mutant is the result of both a decrease in phosphorylation of wt‐PKAα substrates and an increase in the phosphorylation of new, alternative substrates for the L205R mutant.

Surprisingly, the kinetics analysis also shows that the L205R mutation affects the kinetics of the ATP substrate, even though the structural biology data 10 show no conformational change in this binding pocket. Our kinetics data clearly indicate that there is communication between the ATP‐binding site and the peptide substrate‐binding site, as the L205R mutation does affect ATP kinetics. We found that the *K*_M,ATP_ for the L205R‐PKACα mutant was ~ 1.6‐fold higher than for wt‐PKACα. Again, while *K*_M_ is generally viewed as a measure of affinity (*K*_d_), this is an assumption as the expression for *K*_M_ [(*k*_−1_ + *k*_2_)/*k*_1_] contains both the *K*_d_ and the first‐order rate constant for conversion of the [E]·[S] complex to product. As it is unlikely that the affinity of ATP for the binding site of the L205R mutant has changed (based on the structural biology data and our H89 inhibition data), the increase in *K*_M,ATP_ is likely the result of decreased catalysis by the mutant due to a decrease in the binding of the Kemptide substrate. The turnover number data support this, as the *k*_cat,ATP_ for the L205R mutant was ~ 2.8‐fold lower than for wt‐PKACα. However, without additional data on the microscopic rate constants *k*_1_, *k*_−1_, and *k*_2_, we cannot determine whether *k*_cat,ATP_ = *k*_2_ and the exact cause of the decrease in *K*_M,ATP_. For ATP, we again see that the increase in the *K*_M,ATP_ and decrease in *k*_cat,ATP_ appear to reinforce each other, leading to a 4.5‐fold decrease in *k*_cat_/*K*_M_ for ATP. Similar to the results with Kemptide, this decrease in *k*_cat_/*K*_M_ for ATP supports a model for the L205R‐PKACA mutant where the [E]·[S] complex is either dissociating faster than it is being converted to product or that if it is converted to product, catalysis is slower. We cannot, however, rule out that the L205R mutation slows catalysis by affecting the known rate‐limiting step of ADP dissociation 14; additional experimental data would be needed to examine that hypothesis.

Here, we also report the first studies on the susceptibility of the L205R‐PKACα mutant to known inhibitors of wt‐PKACα that determine the fundamental inhibition parameters of IC_50_ and *K*_i_. The L205R mutation has a profound effect on inhibition of the mutant by PKI(5–24), a peptide substrate‐competitive inhibitor (see Table 2 for sequence), with the IC_50_ increasing by > 250‐fold and the *K*_i_ increasing by > 330‐fold. While it is not surprising that the L205R mutation disrupts the binding of PKI(5–24), the loss of potency was larger than we anticipated. We had expected inhibition of L205R‐PKACα with PKI(5–24) to have a *K*_i_ similar to the inhibition of wt‐PKACα with the I22G‐PKI(5–22) inhibitor (*K*_i_ = 470 nm) reported by Walsh and coworkers 15. In the I22G‐PKI(5–22) inhibitor, Ile22 (which binds in the P+1‐binding site) is replaced by Gly and this inhibitor loses the binding affinity from the interaction of the hydrophobic Ile22 side chain with the P + 1 pocket. However, our data for the L205R‐PKACα mutant with PKI(5–24) suggest that the loss of potency may come from more than just loss of the hydrophobic binding interaction in the P + 1 pocket and that there may be some repulsive interactions between the positively charged R205 and the positively charged H23 residue of the C terminus of the PKI(5–24) inhibitor. The ATP‐competitive inhibitor H89, however, displayed nearly identical inhibition of both L205R‐PKACα and wt‐PKACα, as expected from the structural similarity of the ATP‐binding pockets in the crystal structures 10.

The inhibition data that we report here for the L205R mutant have profound implications for the design and development of selective inhibitors of the mutant as potential therapeutics. The standard approach for developing pharmacological inhibitors for kinases in both industry and academia is to use ATP‐competitive small molecules 16. Our finding that the ATP‐competitive inhibitor H89 has equal potency against both the mutant and wt‐PKACα will likely prevent the use of this approach. Any inhibitor for therapeutic use against the mutant will have to be selective for the mutant over wt‐PKACα, in order to not suffer from off‐target toxicity associated with wt‐PKACα inhibition. wt‐PKACα is the primary intracellular signaling kinase for over 75 different G‐protein‐coupled receptors 6 and any potential inhibitor of the L205R mutant that also inhibits wt‐PKACα will likely cause off‐target effects in multiple organ systems and signaling pathways. The previously reported crystallography data for the L205R‐PKACα mutant 10 also support that there will be poor selectivity between wt‐ and L205R‐PKACα, as the ATP‐binding pockets of both enzymes have an identical sequence and, at least in the static crystal form, no conformational differences are seen between wt‐ and L205R‐PKACα. It is possible that a type II kinase inhibitor, which flips the DFG motif into the inactive conformation, could be developed to target the L205R mutant. However, it is unclear whether a type II inhibitor could selectively bind to the mutant over wt‐PKACα. The L205R mutation occurs in the peptide substrate‐binding site, and it is likely that a successful mutant‐selective inhibitor will need to be targeted to that binding site.

## Author contributions

KCE conceived and designed the study. KCE designed the expression plasmid for the recombinant L205R protein. NML and DLP expressed and purified the recombinant L205R protein. CEL performed the trypsin digest and mass spectrometry experiment. NML performed the kinetics and inhibition assays. NML and KCE conducted the data analysis. KCE and NML wrote and edited the manuscript. All authors reviewed the results and approved the final version of the manuscript.

## Supporting information


**Fig. S1.** Recombinant plasmid constructed for L205R‐PKACα.
