# Supplementary material for: Kinetics and inhibition studies of the L205R mutant of cAMP‐dependent protein kinase involved in Cushing's syndrome
Source: FEBS Open Bio. 2018 Mar 11;8(4):606–13. doi: 10.1002/2211-5463.12396 (PMC5881547; doi:10.1002/2211-5463.12396)
Supplement: Supplementary file 1 — Fig. S1. Recombinant plasmid constructed for L205R‐PKACα. [file FEB4-8-606-s001.pdf]

Supplementary Information

Plasmid for expression of recombinant human L205R-PKACα in *E. coli*

Backbone Vector: pET-17b
5'-Cloning Site: NdeI
3'-Cloning Site: HindIII

Key:
5'-NdeI restriction site; $(His)_6$ tag; TEV Cleavage site; PRKACA gene; codon for L205R (T617G); stop codons; 3'-HindIII restriction site

Gene Insert (prepared by synthesis by GenScript):

```
CATATGCACCACCATCACCATCACGAGAATCTGTACTTTCAAGGCAACGCCGCCGCCGCCAAGA
AGGGCAGCGAGCAGGAGAGCGTGAAAGAATTCTTAGCCAAAGCCAAAGAAGATTTTCTTAAAAA
ATGGGAAAGTCCCGCTCAGAACACAGCCCACTTGGATCAGTTTGAACGAATCAAGACCCTCGGC
ACGGGCTCCTTCGGGCGGGTGATGCTGGTGAAACACAAGGAGACCGGGAACCACTATGCCATGA
AGATCCTCGACAAACAGAAGGTGGTGAAACTGAAACAGATCGAACACACCCTGAATGAAAAGCG
CATCCTGCAAGCTGTCAACTTTCCGTTCCTCGTCAAACTCGAGTTCTCCTTCAAGGACAACTCA
AACTTATACATGGTCATGGAGTACGTGCCCGGCGGGGAGATGTTCTCACACCTACGGCGGATCG
GAAGGTTCAGTGAGCCCCATGCCCGTTTCTACGCGGCCCAGATCGTCCTGACCTTTGAGTATCT
GCACTCGCTGGATCTCATCTACAGGGACCTGAAGCCGGAGAATCTGCTCATTGACCAGCAGGGC
TACATTCAGGTGACAGACTTCGGTTTCGCCAAGCGCGTGAAGGGCCGCACTTGGACCTTGTGCG
GCACCCCTGAGTACCGGGCCCCTGAGATTATCCTGAGCAAAGGCTACAACAAGGCCGTGGACTG
GTGGGCCCTGGGGGTTCTTATCTATGAAATGGCCGCTGGCTACCCGCCCTTCTTCGCAGACCAG
CCCATCCAGATCTATGAGAAGATCGTCTCTGGGAAGGTGCGCTTCCCTTCCCACTTCAGCTCTG
ACTTGAAGGACCTGCTGCGGAACCTCCTGCAGGTAGATCTCACCAAGCGCTTTGGGAACCTCAA
GAATGGGGTCAACGATATCAAGAACCACAAGTGGTTTGCCACAACTGACTGGATTGCCATCTAC
CAGAGGAAGGTGGAAGCTCCCTTCATACCAAAGTTTAAAGGCCCTGGGGATACGAGTAACTTTG
ACGACTATGAGGAAGAAGAAATCCGGGTCTCCATCAATGAGAAGTGTGGCAAGGAGTTTTCTGA
GTTTTAATGAAAGCTT
```

Complete Plasmid Sequence (molecular biology performed by GenScript):

```
TTCTTGAAGACGAAAGGGCCTCGTGATACGCCTATTTTTATAGGTTAATGTCATGATAATAATG
GTTTCTTAGACGTCAGGTGGCACTTTTCGGGGAAATGTGCGCGGAACCCCTATTTGTTTATTTT
TCTAAATACATTCAAATATGTATCCGCTCATGAGACAATAACCCTGATAAATGCTTCAATAATA
TTGAAAAAGGAAGAGTATGAGTATTCAACATTTCCGTGTCGCCCTTATTCCCTTTTTTGCGGCA
TTTTGCCTTCCTGTTTTTGCTCACCCAGAAACGCTGGTGAAAGTAAAAGATGCTGAAGATCAGT
TGGGTGCACGAGTGGGTTACATCGAACTGGATCTCAACAGCGGTAAGATCCTTGAGAGTTTTCG
CCCCGAAGAACGTTTTCCAATGATGAGCACTTTTAAAGTTCTGCTATGTGGCGCGGTATTATCC
CGTGTTGACGCCGGGCAAGAGCAACTCGGTCGCCGCATACACTATTCTCAGAATGACTTGGTTG
AGTACTCACCAGTCACAGAAAAGCATCTTACGGATGGCATGACAGTAAGAGAATTATGCAGTGC
TGCCATAACCATGAGTGATAACACTGCGGCCAACTTACTTCTGACAACGATCGGAGGACCGAAG
GAGCTAACCGCTTTTTTGCACAACATGGGGGATCATGTAACTCGCCTTGATCGTTGGGAACCGG
AGCTGAATGAAGCCATACCAAACGACGAGCGTGACACCACGATGCCTGCAGCAATGGCAACAAC
GTTGCGCAAACTATTAACTGGCGAACTACTTACTCTAGCTTCCCGGCAACAATTAATAGACTGG
```

ATGGAGGCGGATAAAGTTGCAGGACCACTTCTGCGCTCGGCCCTTCCGGCTGGCTGGTTTATTG
CTGATAAATCTGGAGCCGGTGAGCGTGGGTCTCGCGGTATCATTGCAGCACTGGGGCCAGATGG
TAAGCCCTCCCGTATCGTAGTTATCTACACGACGGGGAGTCAGGCAACTATGGATGAACGAAAT
AGACAGATCGCTGAGATAGGTGCCTCACTGATTAAGCATTGGTAACTGTCAGACCAAGTTTACT
CATATATACTTTAGATTGATTTAAAACTTCATTTTTAATTTAAAAGGATCTAGGTGAAGATCCT
TTTTGATAATCTCATGACCAAAATCCCTTAACGTGAGTTTTCGTTCCACTGAGCGTCAGACCCC
GTAGAAAAGATCAAAGGATCTTCTTGAGATCCTTTTTTTCTGCGCGTAATCTGCTGCTTGCAAA
CAAAAAAACCACCGCTACCAGCGGTGGTTTGTTTGCCGGATCAAGAGCTACCAACTCTTTTTCC
GAAGGTAACTGGCTTCAGCAGAGCGCAGATACCAAATACTGTCCTTCTAGTGTAGCCGTAGTTA
GGCCACCACTTCAAGAACTCTGTAGCACCGCCTACATACCTCGCTCTGCTAATCCTGTTACCAG
TGGCTGCTGCCAGTGGCGATAAGTCGTGTCTTACCGGGTTGGACTCAAGACGATAGTTACCGGA
TAAGGCGCAGCGGTCGGGCTGAACGGGGGGTTCGTGCACACAGCCCAGCTTGGAGCGAACGACC
TACACCGAACTGAGATACCTACAGCGTGAGCTATGAGAAAGCGCCACGCTTCCCGAAGGGAGAA
AGGCGGACAGGTATCCGGTAAGCGGCAGGGTCGGAACAGGAGAGCGCACGAGGGAGCTTCCAGG
GGGAAACGCCTGGTATCTTTATAGTCCTGTCGGGTTTCGCCACCTCTGACTTGAGCGTCGATTT
TTGTGATGCTCGTCAGGGGGGCGGAGCCTATGGAAAAACGCCAGCAACGCGGCCTTTTTACGGT
TCCTGGCCTTTTGCTGGCCTTTTGCTCACATGTTCTTTCCTGCGTTATCCCCTGATTCTGTGGA
TAACCGTATTACCGCCTTTGAGTGAGCTGATACCGCTCGCCGCAGCCGAACGACCGAGCGCAGC
GAGTCAGTGAGCGAGGAAGCGGAAGAGCGCCTGATGCGGTATTTTCTCCTTACGCATCTGTGCG
GTATTTCACACCGCATATATGGTGCACTCTCAGTACAATCTGCTCTGATGCCGCATAGTTAAGC
CAGTATACACTCCGCTATCGCTACGTGACTGGGTCATGGCTGCGCCCCGACACCCGCCAACACC
CGCTGACGCGCCCTGACGGGCTTGTCTGCTCCCGGCATCCGCTTACAGACAAGCTGTGACCGTC
TCCGGGAGCTGCATGTGTCAGAGGTTTTCACCGTCATCACCGAAACGCGCGAGGCAGCTGCGGT
AAAGCTCATCAGCGTGGTCGTGAAGCGATTCACAGATGTCTGCCTGTTCATCCGCGTCCAGCTC
GTTGAGTTTCTCCAGAAGCGTTAATGTCTGGCTTCTGATAAAGCGGGCCATGTTAAGGGCGGTT
TTTTCCTGTTTGGTCACTGATGCCTCCGTGTAAGGGGGATTTCTGTTCATGGGGGTAATGATAC
CGATGAAACGAGAGAGGATGCTCACGATACGGGTTACTGATGATGAACATGCCCGGTTACTGGA
ACGTTGTGAGGGTAAACAACTGGCGGTATGGATGCGGCGGGACCAGAGAAAAATCACTCAGGGT
CAATGCCAGCGCTTCGTTAATACAGATGTAGGTGTTCCACAGGGTAGCCAGCAGCATCCTGCGA
TGCAGATCCGGAACATAATGGTGCAGGGCGCTGACTTCCGCGTTTCCAGACTTTACGAAACACG
GAAACCGAAGACCATTCATGTTGTTGCTCAGGTCGCAGACGTTTTGCAGCAGCAGTCGCTTCAC
GTTCGCTCGCGTATCGGTGATTCATTCTGCTAACCAGTAAGGCAACCCCGCCAGCCTAGCCGGG
TCCTCAACGACAGGAGCACGATCATGCGCACCCGTGGCCAGGACCCAACGCTGCCCGAGATCTC
GATCCCGCGAAATTAATACGACTCACTATAGGGAGACCACAACGGTTTCCCTCTAGAAATAATT
TTGTTTAACTTTAAGAAGGAGATATACATATGCACCACCATCACCATCACGAGAATCTGTACTT
TCAAGGCAACGCCGCCGCCGCCAAGAAGGGCAGCGAGCAGGAGAGCGTGAAAGAATTCTTAGCC
AAAGCCAAAGAAGATTTTCTTAAAAAATGGGAAAGTCCCGCTCAGAACACAGCCCACTTGGATC
AGTTTGAACGAATCAAGACCCTCGGCACGGGCTCCTTCGGGCGGGTGATGCTGGTGAAACACAA
GGAGACCGGGAACCACTATGCCATGAAGATCCTCGACAAACAGAAGGTGGTGAAACTGAAACAG
ATCGAACACACCCTGAATGAAAAGCGCATCCTGCAAGCTGTCAACTTTCCGTTCCTCGTCAAAC
TCGAGTTCTCCTTCAAGGACAACTCAAACTTATACATGGTCATGGAGTACGTGCCCGGCGGGGA
GATGTTCTCACACCTACGGCGGATCGGAAGGTTCAGTGAGCCCCATGCCCGTTTCTACGCGGCC
CAGATCGTCCTGACCTTTGAGTATCTGCACTCGCTGGATCTCATCTACAGGGACCTGAAGCCGG
AGAATCTGCTCATTGACCAGCAGGGCTACATTCAGGTGACAGACTTCGGTTTCGCCAAGCGCGT
GAAGGGCCGCACTTGGACCTTGTGCGGCACCCCTGAGTACCGGGCCCCTGAGATTATCCTGAGC
AAAGGCTACAACAAGGCCGTGGACTGGTGGGCCCTGGGGGTTCTTATCTATGAAATGGCCGCTG
GCTACCCGCCCTTCTTCGCAGACCAGCCCATCCAGATCTATGAGAAGATCGTCTCTGGAAGGT
GCGCTTCCCTTCCCACTTCAGCTCTGACTTGAAGGACCTGCTGCGGAACCTCCTGCAGGTAGAT

CTCACCAAGCGCTTTGGGAACCTCAAGAATGGGGTCAACGATATCAAGAACCACAAGTGGTTTG
CCACAACTGACTGGATTGCCATCTACCAGAGGAAGGTGGAAGCTCCCTTCATACCAAAGTTTAA
AGGCCCTGGGGATACGAGTAACTTTGACGACTATGAGGAAGAAGAAATCCGGGTCTCCATCAAT
GAGAAGTGTGGCAAGGAGTTTTCTGAGTTTTAATGAAAGCTTGGTACCGAGCTCGGATCCACTA
GTAACGGCCGCCAGTGTGCTGGAATTCTGCAGATATCCATCACACTGGCGGCCGCTCGAGCAGA
TCCGGCTGCTAACAAAGCCCGAAAGGAAGCTGAGTTGGCTGCTGCCACCGCTGAGCAATAACTA
GCATAACCCCTTGGGGCCTCTAAACGGGTCTTGAGGGGTTTTTTGCTGAAAGGAGGAACTATAT
CCGGATAA

Sequence of the Recombinant Protein expressed by the Plasmid:

HHHHHHENLYFQGNAAAAKKGSEQESVKEFLAKAKEDFLKKWESPAQNTAHLDQFERIKTLGTG
SFGRVMLVKHKETGNHYAMKILDKQKVVKLKQIEHTLNEKRILQAVNFPFLVKLEFSFKDNSNL
YMVMEYVPGGEMFSHLRRIGRFSEPHARFYAAQIVLTFEYLHSLDLIYRDLKPENLLIDQQGYI
QVTDFGFAKRVKGRTWTLCGTPEYRAPEIILSKGYNKAVDWWALGVLIYEMAAGYPPFFADQPI
QIYEKIVSGKVRFPSHFSSDLKDLLRNLLQVDLTKRFGNLKNGVNDIKNHKWFATTDWIAIYQR
KVEAPFIPKFKGPGDTSNFDDYEEEEIRVSINEKCGKEFSEF

Note: TEV protease cuts the ENLYFQG sequence between Q and G, leaving G as the first amino acid in the PKACα protein.

Sequence of the Recombinant Protein after TEV protease cleavage (MW = 40,501.5 Da):

GNAAAAKKGSEQESVKEFLAKAKEDFLKKWESPAQNTAHLDQFERIKTLGTGSFGRVMLVKHKE
TGNHYAMKILDKQKVVKLKQIEHTLNEKRILQAVNFPFLVKLEFSFKDNSNLYMVMEYVPGGEM
FSHLRRIGRFSEPHARFYAAQIVLTFEYLHSLDLIYRDLKPENLLIDQQGYIQVTDFGFAKRVK
GRTWTLCGTPEYRAPEIILSKGYNKAVDWWALGVLIYEMAAGYPPFFADQPIQIYEKIVSGKVR
FPSHFSSDLKDLLRNLLQVDLTKRFGNLKNGVNDIKNHKWFATTDWIAIYQRKVEAPFIPKFKG
PGDTSNFDDYEEEEIRVSINEKCGKEFSEF
